# Prevalence and risk factors of deep venous thrombosis of hospitalizations in plateau: a cross-section analysis

**DOI:** 10.1186/s13019-024-02878-6

**Published:** 2024-07-13

**Authors:** Lijuan Sun, Shiqin Pan, Yuemei Li, Mingqin Luo, Xiaofang Li, Hongmei Ma, Jingni Zhang, Limei Wang, Cuo Yong

**Affiliations:** 1 Department of Nursing, Qinghai Provincial People’s Hospital, No.2 Gonghe Road, Xining, 810000 China; 2 Department of Nursing, The Third People’s Hospital of Xining, Xining, 810006 China; 3 Department of Nursing, Yushu People’s Hospital, Yushu, 815000 China

**Keywords:** Deep venous thrombosis, Plateau, Prevalence, Risk factors

## Abstract

**Background:**

Deep venous thrombosis (DVT) is a serious public health issue that threatens human health and economic development. Presently, differences in the prevalence of DVT among individuals from different nationalities, residents of high-altitude areas, and those consuming any special diet are unknown. Therefore, we aimed to elucidate the prevalence of and the associated risk factors for DVT in hospitalized patients in the plateau areas.

**Methods:**

The subjects were hospitalized patients in three grade III-a hospitals in the Qinghai Province, China, during January–October 2020. The demographic, clinical, and laboratory data were collected at admission, and ultrasonography of the bilateral lower extremities was performed. The hospital stay-duration was recorded at the time of discharge.

**Results:**

A total of 3432 patients were enrolled, of which 159 (4.60%) were diagnosed with DVT. The age of > 50 years (OR = 2.434, 95% CI: 1.521–3.894252, *P* < 0.001), residence altitude of ≥ 3000 m (OR = 2.346, 95% CI: 1.239–4.440, *P* = 0.009), D-dimer level of ≥ 0.5 mg/L (OR = 2.211, 95% CI: 1.547–3.161, *P* < 0.001), presence of comorbidities (OR = 1.904, 95% CI: 1.386–2.705, *P* < 0.001), a history of varicose veins (OR = 1.990, 95% CI: 0.959–4.128, *P* = 0.045), and current medications (OR = 2.484, 95% CI: 1.778–3.471, *P* < 0.001) were identified as risk factors for DVT in these plateau areas.

**Conclusion:**

The prevalence of DVT in the hospitalized patients of the studied plateau areas was 4.60%. We recommend considering individualized risk stratification (age > 50 years, residence altitude ≥ 3000 m, a history of varicose veins, D-dimer level ≥ 0.5 mg/L, current medications, and comorbidities) for patients at the time of admission.

## Background

Deep venous thrombosis (DVT) is a venous return disorder caused by abnormal blood clotting in deep veins, and it usually affects the lower extremities. Thrombus migration can cause pulmonary embolism (PE) [1, 2], which can significantly affect the quality of life and even lead to death [3, 4]. The incidence of DVT is 88–112/100 000 person-years [5], and the total incidence of DVT among adults is 50–100/100 000 persons [6]. The most serious complication of DVT is PE, which has been associated with 50,000–200,000 deaths every year [7]. As such, DVT has become a serious public health issue that threatens human health and economic development.

Approximately 500 million people worldwide live at high altitudes [8]. At such altitudes, the air is thin, oxygen partial pressure is low, and the body is in an obvious state of hypoxia. Therefore, the prevalence of DVT is significantly higher in these areas compared to that in plain areas [9, 10]. A past study reported that lowlanders living at high altitudes have a 30–44-times higher risk of presenting to hospitals with DVT than those living at low altitudes [11]; however, data on the prevalence of DVT in high-altitude hospitalized patients are inadequate.

The main causes of DVT are slow blood flow, vessel wall injury, and hypercoagulability. The established risk factors include a history of DVT, presence of a malignant transformation, increasing age, cigarette smoking, obesity, prolonged bed rest, trauma or fracture, surgery, pregnancy and puerperium, use of oral contraceptives and hormone therapy, and general anesthesia [4, 6]. Exposure to high altitudes and hypoxia are the recognized predisposing factors for venous thrombosis [2, 4, 12]. Furthermore, increased red blood cell and blood viscosity [8, 13], platelet aggregation and coagulation activation [4, 14], and vascular endothelial cell damage caused by hypoxia increase the risk of thrombosis in high-altitude hospitalized patients [2, 9, 13, 14]; these reports suggest the presence of other risk factors in plateau areas, warranting further research.

Qinghai province is located in the Qinghai–Tibet Plateau and has an average elevation of approximately 3500 m above sea level. It is a multiethnic region; in addition to the Han nationality, people from many other ethnic minorities such as Tibetan, Hui, Salar, Tu, and Mongolian also live here. These people’s diet is especially high in salt and fat. Presently, no studies have determined whether there are differences in the prevalence of DVT among different nationalities and residence altitudes and whether any special diet culture affects DVT formation. Therefore, it is important to understand the effect of ethnic characteristics, different altitudes, and special diet culture on DVT formation in plateau areas. The present study aimed to investigate the prevalence of DVT in hospitalized patients in plateau areas as well as analyze its risk factors to provide a basis for DVT prevention in plateau areas and also establish a risk assessment system for DVT in plateau areas.

## Methods

### Participants

In this cross-sectional study, we enrolled patients who were hospitalized between January 1, 2020, and December 31, 2020, in three third-class A hospitals in the Qinghai-Tibet Plateau using the following inclusion criteria: [1] patients residing in Qinghai for ≥ 10 years [2], those who were ≥ 18 years of age, and [3] those who voluntarily agreed to participate after providing written informed consent. The exclusion criteria were as follows: [1] patients with difficulty in language communication or understanding [2], those with disturbance of consciousness and who were unable to communicate effectively; further, their caregivers were unable to provide effective information, and [3] patients who were in emergency treatment and died within 24 h after admission. All patients in this study provided their written informed consent. The study was approved by the ethics committee of Qinghai Provincial People’s Hospital (2020-48). All procedures involving human participants were conducted according to the ethical standards of the institutional and/or national research committee and the 1964 Helsinki Declaration and its later amendments or comparable ethical standards.

### Sample size

According to the empirical criteria, the sample size for logistic regression analysis should be 10–15 times the covariate numbers. Accordingly, this study considered 26 covariates; therefore, the sample size was 260–390. Considering 20% of sample shedding, the sample size was ensured to be not less than 468.

### DVT diagnosis

Duplex ultrasonography (DUS) was performed to diagnose DVT. All participants underwent DUS of bilateral lower extremities at the time of admission, and all patients were collected at the same time-point for DUS. We use data from routine DUS. The diagnostic criteria for DVT were noncompressed veins, lumen obstruction or filling defect, lack of respiratory vibration above the knee vein segment, and inadequate flow augmentation to the calves [15]. DUS included the common femoral vein, superficial femoral and deep femoral, popliteal, anterior tibial, posterior tibial, and common fibular veins. Blood clots located in the intermuscular veins (i.e., the gastrocnemius and soleal veins) were not included.

### Data collection

The data collected were demographic variables (age/sex/nationality/residence/body mass index), history of smoking or drinking alcohol, diet consumption (boiled tea/ghee/beef/ mutton), activity ability, diagnoses, comorbidities, family history of DVT, history of varicose veins, current medications, lower limb edema, laboratory examination results (red blood cell count/platelet count/prothrombin time/thrombin time/activated partial thromboplastin time/fibrinogen/D-dimer/fibrinogen degradation products [FDPs]), and total length of hospital stay.

### Statistical analysis

Data analysis was performed using SPSS23.0 (IBM, Armonk, New York, United States). Demographics and measured variables were characterized using descriptive statistics (i.e., mean ± SD, quartile median, and proportions). Continuous variables were analyzed using the Student’s t-test for independent samples or the Mann–Whitney U-test for non-normal distribution variables. Categorical variables were analyzed using the chi-squared test. Multivariate logistic regression analysis was performed to identify the risk factors. The odds ratio (OR) and 95% confidence interval (CI) were used to express the strength of the correlations. *P* < 0.05 was considered to indicate statistical significance.

## Results

### Patient characteristics

In total, 3432 patients were enrolled in this study; of them, 159 (4.60%) patients were diagnosed with DVT. Table 1 lists the characteristics of the study participants.

**Table 1:** Patients characteristics (*n* = 3432)

Particulars	Mean (SD)	Median (P25, P75)	*n* (%)
**Age (year)**	59.94 ±15.55		
**Residence (year)**	56.34 ±17.60		
**Body mass index BMI (kg/m** ^**2**^ **)**	24.21 ±3.89		
Red blood cell count (*10 ^**12**^ **/L)**	4.42 ±2.86		
Platelet count (*10 ^**9**^ **)**	24.81 ±20.43		
**Prothrombin time (PT) (s)**	11.32 ±4.56		
**Thrombin time TT (s)**	16.56 ±3.07		
**Activated partial thromboplastin time APTT (s)**	28.53 ±6.43		
**Fibrinogen, FIB (g/L)**		2.00 (2.00, 3.00)	
**D-Dimer (mg/L)**		0.00 (0.00, 1.00)	
**Fibrinogen degradation products FDP (mg/L)**		2.00 (1.00, 6.00)	
**Gender**			
Male			1875(54.6)
Female			1557(45.4)
**Nationality**			
Han			2686(78.3)
Hui			414(12.1)
Zang			269(7.8)
Others			63(1.8)
**Residence attitude**			
<3000 m			3281(95.6)
≥ 3000 m			151(4.4)
**Smoking history**			
No			2486(72.4)
Yes			946(27.6)
**Alcohol consumption**			
No			2618(76.3)
Yes			814(23.7)
**Boil tea consumption**			
Never			692(20.1)
Occasionally			2193(63.9)
Sometimes			249(7.3)
Frequently			298(8.7)
**Ghee consumption**			
Never			2665(77.6)
Occasionally			489(14.3)
Sometimes			107(3.1)
Frequently			171(5.0)
**Beef and mutton consumption**			
Never			97(2.8)
Occasionally			722(21.1)
Sometimes			1913(55.7)
Frequently			700(20.4)
**Activity ability**			
Paralysis			5(0.2)
Immobilization			41(1.2)
Bedridden ≥ 3 days			313(9.1)
Wheelchair			191(5.6)
Limited activity requires help from Others			327(9.5)
Walking aid			158(4.6)
Autonomic activities			2397(69.8)
**Diagnosis**			
Endocrine, nutritional, metabolic, and immune diseases			872(25.4)
Neurological			129(3.8)
Respiratory			288(8.4)
Digestive			67(1.9)
Circulation			570(16.6)
Genitourinary			63(1.8)
Hematological			12(0.5)
Musculoskeletal system and connective tissue			612(17.8)
Trauma and toxicosis			674(19.6)
Neoplastic			56(1.6)
Others			89(2.6)
**Comorbidities**			
No			2294(66.8)
Yes			1138(33.2)
**DVT family history**			
No			3392(98.8)
Yes			40(1.2)
**History of varicose veins**			
No			3339(97.3)
Yes			93(2.7)
**Current medications**			
No			2547(74.2)
Yes			885(25.8)
**Lower Limb Edema**			
No			137(3.9)
Yes			3295(96.1)

### Differences in the characteristics of patients without and with DVT

Compared with patients without DVT, those with DVT had a longer total length of hospital stay (16.40 ± 7.66 vs. 14.77 ± 8.55, *P* < 0.05). Tables 2 and 3 present the comparison results of the characteristics of the patients without and with DVT. Significant differences were observed in 11 variables: D-dimer levels, FDPs, sex, age, residence altitude, alcohol drinking, activity ability, diagnosis, comorbidities, history of varicose veins, and current medications.

**Table 2:** Comparison between non-DVT and with-DVT in all collected variables

	Non-DVT(*n* = 3273)	With-DVT(*n* = 159)	t/Z	*P*
Total of hospital stay (days)	14.77 ± 8.55	16.40 ± 7.66	-2.609	0.010
BMI (kg/m^2^)	24.22 ± 3.87	24.06 ± 4.40	0.465	0.643
Red blood cells	4.42 ± 2.83	4.39 ± 3.44	0.118	0.906
Platelets	24.82 ± 20.42	24.64 ± 20.60	0.111	0.912
PT (s)	11.31 ± 4.63	11.48 ± 2.57	-0.750	0.454
TT (s)	16.55 ± 2.95	16.67 ± 4.74	-0.329	0.743
APTT (s)	28.51 ± 6.43	28.93 ± 6.26	-0.832	0.406
FIB (s)	2.00 (1.00, 5.00)	5.00 (1.75, 12.25)	-1.246	0.213
D-Dimer (mg/L)	0.00 (0.00, 1.00)	1.00 (0.00, 4.00)	-5.866	<0.001
FDP (mg/L)	2.00 (2.00, 3.00)	3.00 (2.00, 3.00)	-4.626	<0.001

*BMI* body mass index, *RBC* red blood cell, *PT* prothrombin time, *TT* thrombin time, *APTT* activated partial thromboplastin time, *FIB* fibrinogen, *FDP* fibrinogen-degradation product

**Table 3:** Comparison between non-DVT and with-DVT in all collected variables

		Non-DVT(*n* = 3273), *N*(%)	With-DVT(*n* = 159), *N*(%)	X^2^	*P*
Gender	Male	1801(96.05)	74(3.95)	4.405	0.036
	Female	1472(94.54)	85(5.46)		
Age (years)	≤ 50	806(97.70)	19(2.30)	13.343	< 0.001
	> 50	2467(94.63)	140(5.37)		
Nationality	Han	2559(95.27)	127(4.73)	4.972	0.174
	Hui	402(97.10)	12(2.90)		
	Zang	254(94.42)	15(5.58)		
	Others	58(92.06)	5(7.94)		
Residence Attitude (m)	< 3000 m	3134(95.52)	147(4.48)	3.927	0.048
	≥ 3000 m	139(92.05)	12(7.95)		
Smoking history	No	2362(95.01)	124(4.99)	2.574	0.109
	Yes	911(96.30)	35(3.70)		
Alcohol drinking	No	2485(94.92)	133(5.08)	5.000	0.025
	Yes	788(96.81)	26(3.19)		
Boil tea	Never	663(95.81)	29(4.19)	0.693	0.875
	Occasionally	2091(95.35)	102(4.65)		
	Sometimes	237(95.18)	12(4.82)		
	Frequently	282(94.63)	16(5.37)		
Ghee	Never	2545(95.50)	120(4.50)	6.671	0.083
	Occasionally	470(96.11)	19(3.89)		
	Sometimes	97(90.65)	10(9.35)		
	Frequently	161(94.15)	10(5.85)		
Beef and mutton	Never	94(96.91)	3(3.09)	3.024	0.388
	Occasionally	687(95.15)	35(4.85)		
	Sometimes	1817(94.98)	96(5.02)		
	Frequently	675(96.43)	25(3.57)		
Activity ability	paralysis	5(100.00)	0(0.00)	30.341	<0.001
	Immobilization	35(85.37)	6(14.63)		
	Bedridden ≥ 3 days	295(94.25)	18(5.75)		
	Wheelchair	179(93.72)	12(6.28)		
	Limited activity Requires help from others	298(91.13)	29(8.87)		
	Walking aid	151(95.57)	7(4.43)		
	Autonomic activities	2310(96.37)	87(3.63)		
Diagnosis	Endocrine, nutritional, metabolic, and immune diseases	840(96.33)	32(3.67)	19.499	0.034
	Neurological	126(97.67)	3(2.33)		
	Respiratory	262(90.97)	26(9.03)		
	Digestive	62(92.54)	5(7.46)		
	Circulation	547(95.96)	23(4.04)		
	Genitourinary	59(93.65)	4(6.35)		
	Hematological	11(91.67)	1(8.33)		
	Musculoskeletal System and connective tissues	584(95.42)	28(4.58)		
	Trauma and Toxicosis	642(95.25)	32(4.75)		
	Neoplastic	55(98.21)	1(1.79)		
	Others	85(95.51)	4(4.49)		
Comorbidities	No	2217(96.64)	77(3.36)	25.506	<0.001
	Yes	1056(92.79)	82(7.21)		
DVT family history	No	3236(95.40)	156(4.60)	0.753	0.386
	Yes	37(92.50)	3(7.50)		
History of varicose veins	No	3190(95.54)	149(4.46)	8.103	0.004
	Yes	83(89.25)	10(10.75)		
Current medications	No	2466(96.82)	81(3.18)	47.175	<0.001
	Yes	807(91.19)	78(8.81)		
Lower Limb Edema	No	126(91.97)	11(8.03)	4.3	0.116
	Yes	2939(95.58)	136(4.42)		

### Multivariate analyses of risk factors associated with DVT in hospitalizations in the plateau areas

The 11 variables were entered into the multivariate analysis, and the variables assignment are listed in Table 4. age (>50 years), residence attitude (≥ 3000 m), D-dimer level (≥ 0.5 mg/L), comorbidities, history of varicose veins, and current medications acted as independent risk factors of the DVT in high altitude, and patients with-DVT influenced the total hospital stay duration (Table 4.

**Table 4:** Multivariate analyses of risk factors associated with DVT in hospitalizations in plateau areas

	B	Standard error	Wald X^2^	*P*	OR	95% CI	
						lower	upper
Constant	-4.849	0.573	71.678	0	0.008		
Age (>50 years)	0.890	0.240	13.768	< 0.001	2.434	1.521	3.894
Residence attitude (≥ 3000 m)	0.853	0.326	6.859	0.009	2.346	1.239	4.440
D-dimer (≥ 0.5 mg/L)	0.794	0.182	18.958	< 0.001	2.211	1.547	3.161
Comorbidities	0.661	0.171	15.027	< 0.001	1.904	1.386	2.705
History of varicose veins	0.688	0.372	3.417	0.045	1.990	0.959	4.128
Current medications	0.910	0.171	28.445	< 0.001	2.484	1.778	3.471

## Discussion

In our study, the prevalence of DVT in hospitalized patients residing in the plateau areas was 4.6%; this was much higher than the findings of Loffredo et al.(0.25%) [16], Sun et al.(1.65%) [17], and Law et al.(0.03%) [18]. In 2010, a 9-month prospective study in China revealed that the incidence of DVT in China was 0.9%. The 25 hospitals included in this study were all located in plain areas, which further affirms that the plateau environment plays an important role in DVT formation. Hypoxia can lead to vascular wall damage, dysfunction of vascular endothelial cells covering the surface, and increased D-dimer and activated protein c resistance; furthermore, the body exhibits a state of promoting coagulation. On the other hand, it induces the secretion of Weibel–Palade bodies in vascular endothelial cells [2], which store various thrombus formation-related components. The stimulation of hypoxia can lead to changes in blood composition, including increased red blood cell count and hemocytosis, which increases blood viscosity and decreases blood flow [8], as well as promotes hypercoagulability.

We observed that the prevalence of DVT in inpatients residing at altitudes of ≥ 3000 m was considerably higher than that of those residing in altitudes of ≤ 3000 m (7.95% vs. 4.48%). Tibetans, an ethnic group living in the Tibetan Plateau, mostly reside in altitudes of > 3000 m. In the present study, the prevalence of DVT in hospitalized Tibetan patients was higher than that of other ethnic groups (Tibetan, 5.58%; Han, 4.73%; Hui, 2.90%); this may be one of the reasons for the high incidence of DVT in patients residing at altitudes of ≥ 3000 m. Deng et al. [19] compared the blood components of 222 patients who had been hospitalized in plateau areas and found that the levels of Hb and Hct and RBC count in the blood of the patients increased with an increase in the altitude.

We also observed that the prevalence of DVT in patients > 50 years was significantly higher than that in patients < 50 years (5.37% vs. 2.30%); this is consistent with the findings of Nordstrom et al. [20]. The risk of blood clots is strongly associated with aging [20, 21]. Hb level, Hct level, and RBC count in the blood of permanent residents in high-altitude areas increased with age [22]; further, the D-dimer level increased with age [5]. All these factors may increase the risk of DVT in people > 50 years.

The diagnostic value of D-dimer for thrombus was confirmed in the Guidelines for Diagnosis and Treatment of Acute Pulmonary Embolism in 2019 issued by the European Society of Cardiology [23]; it was further verified in the present study. We found that when patients’ D-dimer level is ≥ 0.5 mg/L, attention should be paid to evaluate the presence of DVT symptoms. The D-dimer level increases with age, leading to a decrease in the specificity of DVT diagnosis in elderly patients [5]. Righini et al. [24] suggest adjusting the D-dimer threshold for patients > 50 years to improve the specificity of DVT diagnosis; this aspect has been verified in related studies [24, 25]. Medical staff should not only pay attention to the D-dimer index of patients but also apply the age-adjusted D-dimer strategy to improve the diagnostic specificity for patients > 50 years [25].

This study indicates that comorbidity is an independent risk factor for DVT in hospitalized patients. Some studies [26–28] have reported that complications increase the thrombosis risk. In this study, we included 33.16% of patients with complications, and the prevalence of DVT was as high as 7.21%. A meta-analysis by Tang et al. [29] revealed that the risk of DVT in patients with stroke and complications of diabetes, coronary heart disease, atrial fibrillation, and heart failure increased by 1.63, 2.31, 1.55, and 1.96 times, respectively. Wei B et al. [30] and Wang S et al. [31] reported that patients with spinal diseases and hypertension, diabetes, and heart disease exhibited an increased risk of postoperative DVT. In patients with renal insufficiency, the risk of thrombosis was reported to increase by 5.5 times [32]. A prospective registry of 5451 patients with DVT by Goldhaber SZ et al. [33] reported that 50% of these patients had hypertension. Therefore, the medical staff should take active and effective preventive measures to minimize the onset of complications. For patients with complications, effective preventive measures for DVT should be taken while strengthening observation and evaluating factors to reduce DVT risk.

The present results suggest that varicose vein history is an independent risk factor for DVT in hospitalized patients. Varicose veins are common in the lower extremities of patients and are among the manifestations of venous insufficiency of the lower extremities. Wołkowski K. et al. have reported that varicose veins of the lower extremities were assessed as a possible risk factor for DVT [34]. A total of 2.71% of patients had varicose veins in this study, and the prevalence of DVT was as high as 10.75%. The incidence of chronic mountain sickness among residents living at altitudes > 2500 m was reported to be 5–10% [35, 36], including varicose veins. Gao et al. [37] studied 218 individuals living at an altitude of 3300–5400 m in 2019, and the findings revealed that the incidence of varicose veins in areas of high altitude was as high as 11.47%. For patients with varicose veins, it is necessary to understand the extent of varicose veins.

Current medications were found to be an independent risk factor for DVT in hospitalized patients. In this study, the proportion of patients taking medications was 25.79%, and the prevalence of DVT was as high as 8.81%. Various drugs are clinically applied. Drugs that have been proven to be likely to cause thrombosis include sedation, dehydration, and vasopressin drugs. These drugs induce hemodynamic changes and vascular damage in patients, among which vasopressin reduces the vascular lumen and increases the risk of thrombosis.

Antibiotics are commonly used to prevent and control infection and are very commonly used in clinical settings. Timp et al. [38] revealed that, with the use of antibiotics, the risk of VTE was 4.5 times higher and that of DVT was 3.2 times higher than that without the use of antibiotics. A population-based case-control study by Schmidt et al. [39] revealed a nearly three-fold increase in the risk of DVT after community antibiotic treatment. The mechanism of cefoperazone/sulbactam-induced coagulation dysfunction may be related to the presence of the N-methylthiotetrazolium side chain in cefoperazone, which possesses a molecular structure similar to that of glutamate. It competitively binds glutamate carboxylase along with vitamin K1 in the liver microsomes. The resulting structure affects the synthesis of vitamin K-dependent coagulation factors II, VII, IX, and X, thereby leading to coagulation dysfunction in the body [40]. Moreover, cefoperazone cannot be efficiently metabolized in the body, and > 40% is discharged from the bile duct through the intestine. Cefoperazone inhibits normal intestinal flora and the intestinal synthesis of vitamin K, resulting in vitamin K-dependent prothrombin hypoemia. These effects result in coagulation dysfunction, which is characterized by prolonged INR, PT, and APTT, as well as a bleeding tendency [41]. Therefore, medical staff should improve antibiotic-related awareness regarding the risk of thrombosis. Furthermore, they should rationally use antibiotics based on the results of drug sensitivity tests and reduce the administration of unnecessary antibiotics to reduce the risk of thrombosis.

The total length of hospital stay of patients with DVT in this study was longer than that of patients without DVT (16.40 ± 7.66 days vs. 14.77 ± 8.55 days, respectively). With an extended hospital stay, the economic burden on the patients, hospitals, and society also increases and brings more risks. Therefore, the prevention and treatment of DVT are particularly important. Thus, we can combine the characteristics of the plateau areas to build a VTE prevention and management system in the plateau areas, improve patient prognoses, improve the quality of medical treatment, and guarantee the medical safety of inpatients to reduce the VTE-related disease and economic burden.

### Strength and limitation

Presently, the data on the prevalence of DVT among the inpatients on the Tibetan Plateau in China is very limited. Through our study findings, we could provide a basis for the prevention and treatment of DVT in the Tibetan Plateau in the future. Nonetheless, there are some limitations to our study. First, the outpatients were not included in this study, which may have underestimated the prevalence of DVT in the plateau areas. Second, the sample size was relatively small considering the large population of people in plateau areas, especially of the Zang, Hui, Sala, and Meng nationalities. Third, we neither classified the proportion of DVT in the various districts (such as femoral and popliteal) nor considered superficial vein thrombosis in this study. Although data on RBC and other coagulation indicators were collected, no statistical differences were noted between the DVT and non-DVT groups. Therefore, in the future, the levels of hemoglobin (Hb) and hematocrit (Hct) should also be considered. Fourth, the ethnic population of Tibet might possess an inborn hypercoagulability, which should be observed in the future.

## Conclusions

Currently, the data on the prevalence of DVT among inpatients on the Tibetan Plateau in China is limited. Our study findings provide a basis for DVT prevention and treatment in the Tibetan Plateau in the future. Based on our findings, we suggest that medical staff should use an appropriate DVT evaluation model to assess DVT-independent risk factors and prevalence. Based on the outcomes, targeted preventive measures along with plateau characteristics can be applied to reduce the DVT prevalence. During hospitalization, it is important that patients focus on assessing the conditions of their lower extremities.

## Data Availability

No datasets were generated or analysed during the current study.

## Abbreviations

DVT: Deep Venous Thrombosis
DUS: Duplex Ultrasonography
FDPs: Fibrinogen Degradation Products
OR: Odds Ratio
CI: Confidence Interval
PT: Prothrombin Time
TT: Thrombin Time
APTT: Activated Partial Thromboplastin Time
FIB: Fibrinogen
